# Prognostic Models for Nonmetastatic Triple-Negative Breast Cancer Based on the Pretreatment Serum Tumor Markers with Machine Learning

**DOI:** 10.1155/2021/6641421

**Published:** 2021-05-15

**Authors:** Huihui Chen, Shijie Wu, Jun Hu, Kun Zhang, Kaimin Hu, Yuexin Lu, Jiapan He, Tao Pan, Yiding Chen

**Affiliations:** ^1^Department of Breast Surgery, The Second Affiliated Hospital, Zhejiang University School of Medicine, 88 Jie-Fang Rd, Hangzhou, Zhejiang 310009, China; ^2^The Key Laboratory of Cancer Prevention and Intervention, China National Ministry of Education, 88 Jie-Fang Rd, Hangzhou, Zhejiang 310009, China; ^3^Department of General Surgery, Hangzhou Fuyang Hospital of Traditional Chinese Medicine, 2-4 Gui-Hua Rd, Fuyang Street, Hangzhou, Zhejiang 311400, China

## Abstract

**Purpose:**

Triple-negative breast cancer (TNBC) is a heterogeneous and aggressive disease with poorer prognosis than other subtypes. We aimed to investigate the prognostic efficacy of multiple tumor markers and constructed a prognostic model for stage I-III TNBC patients. *Patients and Methods*. We included stage I-III TNBC patients whose serum tumor markers levels were measured prior to the treatment. The optimal cut-off value of each tumor marker was determined by X-tile. Then, we adopted two survival models (lasso Cox model and random survival forest model) to build the prognostic model and AUC values of the time-dependent receiver operating characteristic (ROC) were calculated. The Kaplan-Meier method was used to plot the survival curves and the log-rank test was used to test whether there was a significant difference between the predicted high-risk and low-risk groups. We used univariable and multivariable Cox analysis to identify independent prognostic factors and did subgroup analysis further for the lasso Cox model.

**Results:**

We included 258 stage I-III TNBC patients. CEA, CA125, and CA211 showed independent prognostic value for DFS when using the optimal cut-off values; their HRs and 95% CI were as follows: 1.787 (1.056–3.226), 2.684 (1.200–3.931), and 2.513 (1.567–4.877). AUC values of lasso Cox model and random survival forest model were 0.740 and 0.663 for DFS at 60 months, respectively. Both the lasso Cox model and random survival forest model demonstrated excellent prognostic value. According to tumor marker risk scores (TMRS) computed by the lasso Cox model, the high TMRS group had worse DFS (HR = 3.138, 95% CI: 1.711–5.033, *p* < 0.0001) and OS (3.983, 1.637–7.214, *p*=0.0011) than low TMRS group. Furthermore, subgroup analysis of N_0_-N_1_ patients in the lasso Cox model indicated that TMRS still had a significant prognostic effect on DFS (2.278, 1.189–4.346) and OS (2.982, 1.110–7.519).

**Conclusions:**

Our study indicated that pretreatment levels of serum CEA, CA125, and CA211 had independent prognostic significance for TNBC patients. Both lasso Cox model and random survival forest model that we constructed based on tumor markers could strongly predict the survival risk. Higher TMRS was associated with worse DFS and OS both in stage I-III and N_0_-N_1_ TNBC patients.

## 1. Introduction

Breast cancer is the most common malignancy among women throughout the world, with the highest morbidity and mortality in various female cancers. According to the global cancer statistics report released by the World Health Organization, there would be about 2.08 million newly diagnosed female breast cancer cases and more than 0.62 million patients died of it in 2018 [1]. Triple-negative breast cancer (TNBC) is characterized by the absence of estrogen receptor (ER), progesterone receptor (PR) expression, and human epidermal growth factor receptor-2 (HER-2) amplification, accounting for 10%-20% of all breast cancers [2–4]. TNBC patients usually have more unfavorable histopathologic features when compared with non-TNBC, such as more rapid proliferation, larger tumor size, higher grade, and lymph node positivity [5, 6]. TNBC patients can not benefit from endocrine therapy or anti-HER-2 therapy since targets are missing, making chemotherapy become currently the mainstay of systemic treatment.

Notorious for its heterogeneity, aggressiveness, and limited treatment options, TNBC is thought to have the poorest prognosis in all subtypes. Although it is reported that TNBC patients are sensitive to chemotherapy as demonstrated by higher pathologic complete response (pCR) rates than other subtypes after neoadjuvant chemotherapy [7, 8]. There are still a considerable number of patients who cannot obtain pCR, and those with residual lesions have significantly worse survival compared to non-TNBC [7]. On the other hand, there is a higher risk of relapse and disease progression after surgery and chemotherapy for TNBC [9, 10]. Montagna E et al. evaluated the outcome of breast cancer patients after locoregional recurrence (LRR) furtherly and they found that patients with TNBC at LRR experienced a higher risk of subsequent relapse and death [11]. Recently, a retrospective analysis based on the SEER database also revealed that when in comparison with non-TNBC, TNBC patients had worse overall survival (OS) and breast cancer cause-specific survival (BCSS) in every stage and substage [12]. As for the survival of those patients with distant metastasis, it is also shorter in TNBC compared to other subtypes and this can be explained by the predilection for brain and lung metastasis of TNBC, while ER-positive breast cancers are more likely to relapse in bone or skin [4, 13, 14]. Therefore, it is important to discover some efficient and easy detection prognostic markers to evaluate the risk of postoperative recurrence or survival.

Apart from the extensively documented clinicopathological risk factors such as lymph node status, tumor size, grade, and the level of Ki-67, there are still no prognostic biomarkers suitable for clinical use in TNBC [15, 16]. The prognostic value of serum tumor markers has been investigated in breast cancer for several years and carcinoembryonic antigen (CEA) and cancer antigen 15-3 (CA15-3) are the most widely used tumor markers in clinical practice [17–21]. However, the prognostic efficacy of preoperative levels of serum tumor markers such as CEA and CA15-3 in breast cancer remains controversial. Several previous studies suggested that elevated preoperative CEA and CA15-3 levels are associated with tumor burden and poor prognosis [17, 22, 23]. In contrast, there are also some reports that failed to support this conclusion, showing no prognostic significance of CEA or CA15-3 [21, 24]. Although the European Group on Tumor Markers has recommended the use of CEA and CA15-3 for assessing prognosis and early detection of disease progression in breast cancer since 2005 [25], the American Society of Clinical Oncology (ASCO) and National Comprehensive Cancer Network (NCCN) guidelines have not recommended the routine utilization of CEA and CA15-3 [26, 27]. Additionally, most studies have been based on breast cancer overall; the association of these tumor markers and different subtypes of breast cancer such as TNBC remains to be clarified.

In recent years, machine learning methods have been widely applied to disease prognosis and prediction [28–30]. These techniques are utilized for identifying informative factors and modeling the progression of cancer. Park et al. compared three classification models, namely, support vector machines (SVM), artificial neural network (ANN), and semisupervised learning models (SSLM) for the prediction of breast cancer survivability based on 16 features, including tumor size, the number of nodes, and age [28]. However, SVM, ANN, and SSLM, which are designed for classification data, are not suitable for time-to-event data. Lasso Cox regression model and random survival forest model are commonly used survival machine learning algorithms. For example, Zheng et al. developed a novel scoring system based on hypoxia and immune status by taking the lasso Cox regression model [30].

In our study, we intended to conduct research to investigate the prognostic efficacy of multiple tumor markers and constructed prognostic models for stage I-III TNBC patients based on the six pretreatment tumor markers' levels (including CEA, CA19-9, CA125, CA242, CA211, and CA15-3) with machine learning algorithms, so as to help identify the early-stage patients with high recurrence and mortality risk.

## 2. Patients and Methods

### 2.1. Study Population

We conducted a retrospective analysis of stage I-III TNBC patients who were admitted to The Second Affiliated Hospital of Zhejiang University, School of Medicine, between January 2011 and December 2017 and whose serum tumor markers (including CEA, CA19-9, CA125, CA242, CA211, CA15-3) levels were measured prior to surgery or neoadjuvant chemotherapy. TNBC was defined as ER and PR negative or <1% if the percentage was specified and HER-2 status is 0 or 1+ by immunohistochemistry analysis or 2+ with negative fluorescent in situ hybridization [31, 32]. Patients with any missing receptor information or a missing pathology report were excluded from the analysis. In addition, the patients were also excluded for meeting one of the following criteria: (1) carcinoma in situ; (2) male patients; (3) stage IV disease with distant metastasis at first diagnosis; (4) history of other malignant tumors. All data, including clinical and pathological information, treatment modality, serum tumor markers, and details of outcomes, were collected. TNM stage was based on the Eighth American Joint Committee on Cancer Criteria. The written informed consent was acquired from each breast cancer patient or patient's guardian and the study was approved by the Ethics Committee of The Second Affiliated Hospital of Zhejiang University, School of Medicine.

### 2.2. Tumor Markers Detection

Peripheral blood samples (5 mL) were collected from all patients before treatment. Then serum was separated by centrifugation kept at −80°C for later detection. The serum CEA, CA19-9, CA125, CA242, CA211, and CA15-3 levels were measured using the chemiluminescence immunoassay method (ARCHITECT i2000; Abbott Laboratories Inc). The cut-off values for normal and elevated tumor markers were 5 ng/mL for CEA, 37 U/mL for CA19-9, 35 U/mL for CA125, 20 U/mL for CA242, 5 ng/mL for CA211, and 30 U/mL for CA15-3.

### 2.3. Follow-Up and Study Endpoints

Patients were followed up at an interval of 3 months within 2 years, 6 months within 3–5 years, and 1 year for more than 5 years, with the date of surgery performed considered as the first day of follow-up. The primary study endpoints were disease-free survival (DFS) and overall survival (OS). DFS was defined to be from the date of surgery to the date of locoregional recurrence, distant metastasis, another second primary cancer, and death before recurrence or the date of the last follow-up. OS was defined to be from the date of surgery to death from any cause or the date of the last follow-up.

### 2.4. Lasso Cox Model and Random Survival Forest Model

The least absolute shrinkage and selection operator (lasso) Cox regression model analysis was performed by using the “glmnet” package [33]. Partial likelihood deviance was selected as the loss function, and the optimal values of penalty parameter *λ* were determined through twenty-fold cross-validation [34]. Regression coefficients of each tumor marker were calculated with the optimal *λ* value, and tumor marker risk scores (TMRS) of patients were then calculated based on the levels of serum tumor markers and their associated regression coefficients accordingly.

Random survival forest (RSF) is an extension of Breiman's random forest method which was designed for analysis of right-censored time-to-event data [35]. We performed a RSF model to build the predictive model using the “randomForestSRC” package [35]. Tuning parameters, such as node size and mtry, where node size represented the number of samples in the terminal node and mtry was the number of randomly selected candidate variables in each parent node, were optimized by a grid search to minimize the out-of-bag (OOB) error. TMRS of the RSF model were calculated utilizing the “predict” function of the “stats” package. With the median TMRS as a cut-off value, all TNBC patients were split into high TMRS and low TMRS groups in both models.

### 2.5. Statistical Analysis

Statistical evaluation of comparison of each tumor marker levels in different stages was performed using one-way analysis of variance (ANOVA) and Tukey's post hoc test or nonparametric Kruskal-Wallis test according to the distribution and homogeneity test of variances of data. X-tile 3.6.1 software (Yale University, New Haven, CT, USA) was used to determine the optimal prognostic cut-off value of each tumor marker in TNBC patients [36]. The sensitivity and specificity of the survival prediction based on the TMRS were depicted by a time-dependent receiver operating characteristic (ROC) curve, with quantification of the area under the ROC curve (AUC) using the “timeROC” package [37]. All packages were used in our study to analyze data with the R project (version 3.4.2). Graphpad prism 6 was used to plot Kaplan-Meier survival curves and the group differences in survival time were tested using the log-rank test, with hazard ratios (HRs) and 95% confidence intervals (CIs) being calculated. The difference between proportions was evaluated by the chi-square or Fisher's exact test as appropriate. Univariable and multivariable Cox's proportional hazard analyses were performed to compare and identify independent prognostic factors for DFS. All tests were 2-sided and statistical significance was set at *p* < 0.05. All data were analyzed using the SPSS 24.0 and Graphpad prism 6 software.

## 3. Results

### 3.1. Patient Characteristics and Follow-Up

258 stage I-III TNBC patients met the criteria for inclusion in the study. The clinicopathological characteristics of the patients are shown in Table 1. The median age at diagnosis for participants was 51.5 years old (range 25–87 years). Among them, the age of disease onset in most (68.2%) patients was between 40 and 60 years. Bilateral morbidity was basically the same, with left 50.8% and right 48.8%, respectively. One patient was diagnosed with bilateral breast cancer, left invasive ductal carcinoma and right carcinoma in situ, with both sides having a negative expression of ER, PR, and HER-2. The pathological classification of 203 cases (78.7%) was nonspecific invasive cancer. 110 (42.6%) patients were classified as histologic grade III and the expression of Ki-67 was ≥30% (high expression) in 193 cases (74.8%). As for the TNM stage, there were 100 cases (38.8%) in stage I, 111 cases (43.0%) in stage II, and 36 (14.0%) in stage III. In addition, a total of 178 (69.0%) patients underwent a total mastectomy, and 80 (31.0%) received breast-conserving surgery. 236 patients (91.5%) received chemotherapy (including adjuvant and neoadjuvant) and 114 cases (44.2%) received postoperative radiotherapy. During follow-up, 53 patients (20.5%) displayed disease progression, with 16 of locoregional recurrence (6.2%), 31 of distant metastasis (12.0%), 3 of second primary cancer (1.2%), and 3 of death because of other reasons (1.2%). Moreover, 28 patients (10.8%) died, 23 of whom died of breast cancer.

**Figure 1:**
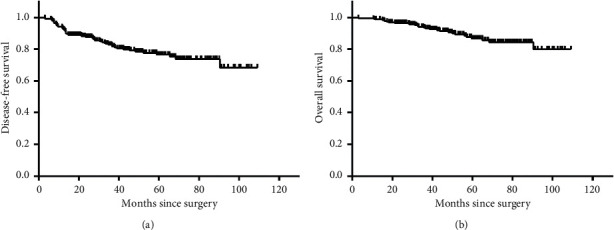
Kaplan-Meier survival curves in all included TNBC patients. (a) DFS. (b) OS. TNBC: triple-negative breast cancer; DFS: disease-free survival; OS: overall survival.

Kaplan-Meier survival curves of DFS and OS in all included TNBC patients are shown in Figure 1. The median follow-up time of our study population was 41.25 months for DFS and 49.25 months for OS. The 5-year DFS and OS were 76.5% and 86.7%, respectively (Figures 1(a) and 1(b)).

### 3.2. The Levels of Pretreatment Serum Tumor Markers

**Figure 2:**
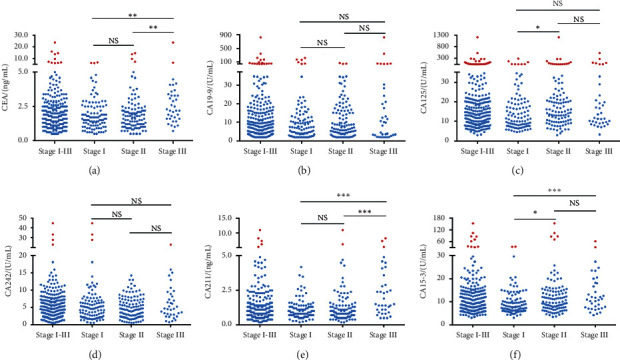
The levels of each serum tumor marker in stage I-III TNBC patients. (a) CEA. (b) CA19-9. (c) CA125. (d) CA242. (e) CA211. (f) CA15-3. A scatter represents a patient, and the cut-off value of each scatter plot is the clinical upper limit, in which higher than the cut-off is indicated by red scatter while the lower is blue. The comparison between different stages was performed using one-way ANOVA and Tukey's post hoc test or the nonparametric Kruskal-Wallis test as appropriate. ^*∗*^*p* < 0.05, ^*∗∗*^*p* < 0.01, ^*∗∗∗*^*p* < 0.001 indicated a significant difference. CEA: carcinoembryonic antigen; CA: cancer antigen; TNBC: triple-negative breast cancer; NS: not significant.

Figure 2 shows the distribution of each tumor marker among different stages patients. First of all, for these early-stage TNBC patients, there were only a few people with elevated serum tumor markers levels. For example, only 10 (3.9%), 17 (6.6%), and 10 (3.9%) patients showed elevated levels of CEA, CA19-9, and CA15-3. However, in the comparison of stage I-III, the elevations of four markers (including CEA, CA125, CA211, and CA15-3) tend to be more found in more advanced stages (stage II or III). As we can see in Figures 2(a) and 2(e), the serum levels of CEA and CA211 were significantly higher in stage III patients than those in stage I and stage II. In terms of CA15-3, both stage II and III TNBC patients showed higher levels than stage I (Figure 2(f)). However, there was no obvious correlation between serum levels of CA19-9, CA242, and TNM stage (Figures 2(b) and 2(d)).

On the other hand, we also compared the levels of tumor markers among patients without recurrence evidence, with locoregional recurrence and with distant metastasis, respectively (Figure S1). The results suggested that for those with different DFS status, their pretreatment levels of serum tumor markers had no significant difference.

### 3.3. The Optimal Cut-Off Values Determined by X-Tile and Their Prognostic Role

Stage II or III patients showed higher levels of tumor markers than stage I patients, but only a few people had elevated tumor markers levels; we did not think it was appropriate to use the clinical cut-off value as the prognostic cut-off for early-stage TNBC patients. So, we used X-tile to determine the optimal prognostic cut-off value of each tumor marker, and as shown in Table 2, the optimal cut-off values of CEA, CA19-9, CA125, CA242, CA211, and CA15-3 were 2.15 ng/mL, 17.30 U/mL, 9.05 U/mL, 8.85 U/mL, 1.15 ng/mL and 16.00 U/mL, respectively.

**Figure 3:**
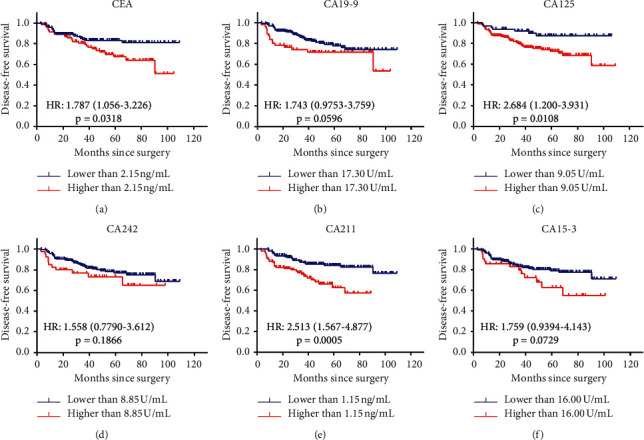
Kaplan-Meier survival curves of TNBC patients DFS based on the optimal cut-off value. (a) CEA. (b) CA19-9. (c) CA125. (d) CA242. (e) CA211. (f) CA15-3. The group differences in survival time were tested using the log-rank test; HRs with 95% CIs and (p) value were shown in the figure. HR and its 95% CI larger than 1 indicated a poorer prognosis of high-level tumor marker. *P* < 0.05 reported the significant differences. TNBC: triple-negative breast cancer; DFS: disease-free survival; CEA : carcinoembryonic antigen; CA: cancer antigen; HR: hazard ratio; CI: confidence interval.

Based on the newly determined cut-off value, we plotted the Kaplan-Meier survival curve of each tumor marker, as shown in Figure 3. Compared with lower tumor makers levels, higher CEA, CA125, and CA211 levels were clearly associated with poor DFS, and their corresponding HRs and 95% CIs were as follows: 1.787 (1.056–3.226), 2.684 (1.200–3.931), and 2.513 (1.567–4.877) (Figures 3(a), 3(c), and 3(e)). As for CA19-9 (HR = 1.743, 95% CI: 0.975–3.759, *p*=0.0596), CA242 (HR = 1.558, 95% CI: 0.779–3.612, *p*=0.1866), and CA15-3 (HR = 1.759, 95% CI: 0.939–4.143, *p*=0.0729), although we still could not find their significantly independent prognostic value, there was a tendency that patients with high levels of serum tumor markers had poorer prognosis (Figures 3(b), 3(d) and 3(f)). Thus, we aimed to evaluate patients' prognosis according to the levels of these six tumor markers.

Construction of the Prognosis Prediction Model for TNBC Patients by Lasso Cox Model and Random Survival Forest Model.

**Figure 4:**
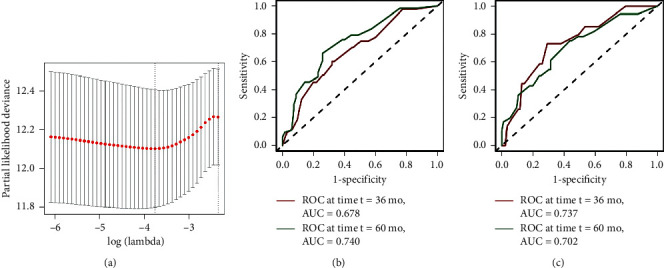
The tuning parameter plot and time-dependent ROC curves of the lasso Cox model. (a) The tuning parameter plot. The *x*-axis represents log-transformed lambda values, and the *y*-axis represents the partial likelihood deviance. The vertical dashed line indicates the minimal partial likelihood deviance. (b) tdROC curve for DFS at 36 months, with an AUC of 0.678 and at 60 months, with an AUC of 0.740. (c) tdROC curve for OS at 36 months, with an AUC of 0.737 and at 60 months, with an AUC of 0.702. tdROC: time-dependent receiver operating characteristic; AUC: area under the ROC curve; DFS: disease-free survival; OS: overall survival.

We counted it as 1 score if the level of each serum tumor marker was higher than the optimal cut-off value, otherwise as 0 score. Based on the levels of these tumor markers, the lasso Cox model identified the risk signature that was significantly associated with DFS based on the optimal *λ* value 0.0234 (Figure 4(a)). The lasso algorithm is a shrinkage estimate that can be used to construct a penalty function and obtain a relatively refined model [34]. Here in our study, the regression coefficient of CA242 turned into zero, while the remaining tumor markers were included in the simplified lasso Cox model (Table 3). TMRS of each patient was then calculated based on these regression coefficients and levels of five tumor markers. Time-dependent ROC curve analysis showed that prognostic accuracy of TMRS was 0.678 at 36 months and 0.740 at 60 months for DFS; 0.737 at 36 months and 0.702 at 60 months for OS (Figures 4(b) and 4(c)).

**Figure 5:**
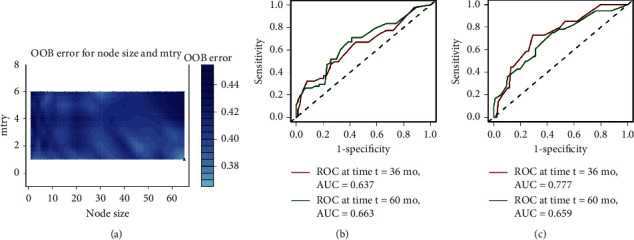
The tuning parameter plot and time-dependent ROC curves of the random survival forest model. (a) The tuning parameter plot. Node size is the number of samples in the terminal node and mtry is the number of randomly selected candidate variables in each parent node; OOB error is the out-of-bag error. A darker color indicates a larger OOB error, while a lighter color indicates smaller OOB error, suggesting a better RSF model. (b) tdROC curve for DFS at 36 months, with an AUC of 0.637 and at 60 months, with an AUC of 0.663. (c) tdROC curve for OS at 36 months, with an AUC of 0.777 and at 60 months, with an AUC of 0.659. RSF: random survival forest; tdROC: time-dependent receiver operating characteristic; AUC: area under the ROC curve; DFS: disease-free survival; OS: overall survival.

We further chose another machine learning method, the RSF model, to build the predictive model. As Figure 5(a) shows, the OOB error was lowest when mtry was 1 and node size was 65, indicating the best RSF model. In this model, the recurrence risk of each patient was computed as well, and time-dependent ROC curves were plotted then. As is shown in Figures 5(b) and 5(c), AUC values were 0.637 and 0.663 at 36 and 60 months for DFS; 0.777 and 0.659 at 36 and 60 months for OS, respectively.

### 3.4. Prognostic Value of TMRS Groups in Two Survival Models and Subgroup Analysis

**Figure 6:**
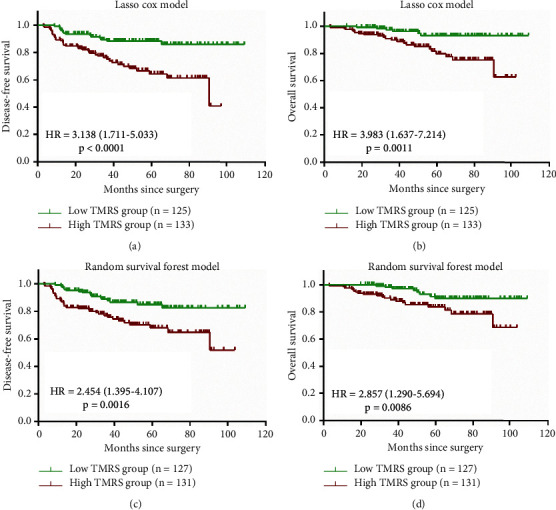
Kaplan-Meier survival curves of lasso Cox and random survival forest model in total TNBC patients. (a) DFS of lasso Cox model in all TNBC patients. (b) OS of lasso Cox model in all TNBC patients. (c) DFS of RSF model in all TNBC patients. (d) OS of RSF model in all TNBC patients. The group differences in survival time were tested using the log-rank test; HRs with 95% CIs and (p) value were shown in the figure. HR and its 95% CI larger than 1 indicated a poorer prognosis of high TMRS. *P* < 0.05 reported the significant differences. TNBC: triple-negative breast cancer; TMRS: tumor marker risk score; HR: hazard ratio; CI: confidence interval; DFS: disease-free survival; OS: overall survival; RSF: random survival forest.

The median TMRS was used as the threshold to divide total TNBC patients into high-risk and low-risk groups in both models and survival analyses of the total study population were performed in terms of TMRS. According to the risk scores calculated by the lasso Cox model, higher TMRS was significantly associated with worse DFS (HR = 3.138, 95% CI: 1.711–5.033, *p* < 0.0001) and OS (HR = 3.983, 95% CI: 1.637–7.214, *p*=0.0011). The 5-year DFS and OS of the low TMRS group vs. the high TMRS group patients were 88.5% vs. 64.4% and 93.3% vs. 79.9%, respectively (Figure 6(a) and 6(b)). On the other hand, the RSF model also showed great predictive value for nonmetastatic TNBC patients. The survival analysis indicated that patients in the high-risk group had significantly higher recurrence risk (HR = 2.454, 95% CI: 1.395–4.107, *p*=0.0016) and mortality risk (HR = 2.857, 95% CI: 1.290–5.694, *p*=0.0086) than those in the low-risk group (Figure 6(c) and 6(d)).

**Figure 7:**
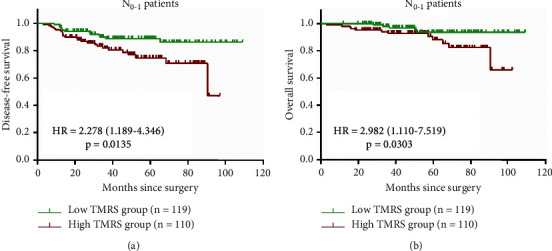
Kaplan-Meier survival curves of lasso Cox model in N_0_-N_1_ TNBC patients. (a) DFS in N_0_-N_1_ TNBC patients. (b) OS in N_0_-N_1_ TNBC patients. The group differences in survival time were tested using the log-rank test; HRs with 95% CIs and (p) value were shown in the figure. HR and its 95% CI larger than 1 indicated a poorer prognosis of high TMRS. *P* < 0.05 reported the significant differences. TNBC: triple-negative breast cancer; TMRS: tumor marker risk score; HR: hazard ratio; CI: confidence interval; DFS: disease-free survival; OS: overall survival.

We further chose the lasso Cox model to evaluate the model performance in the subgroup analysis since it had a larger AUC value and better prognostic significance than the RSF model, with good interpretability for the survival model. Univariable analysis showed that T-stage (*p*=0.093), N-stage (*p* < 0.001) and TMRS groups (*p* < 0.001) were potential prognostic factors for DFS (Table 4). Multivariable analysis including these factors demonstrated that besides TMRS groups, the traditional clinicopathological factor, N-stage, had independent prognostic value for DFS in TNBC patients as well (*p* < 0.001, Table 4). When stratified by lymph node status (N-stage), N_2_-stage (HR = 2.767, 95% CI: 1.218–6.288) and N_3_-stage (HR = 4.980, 95% CI: 2.081–11.917) patients showed poorer prognosis than N_0_-stage patients, while N_1_-stage showed no significant difference (HR = 0.658, 95% CI: 0.263–1.650) (Table 4). Hence, we selected N_0_-N_1_ patients as low recurrence risk patients and plotted the Kaplan-Meier survival curve according to TMRS groups. As in Figures 7(a) and 7(b), TMRS groups showed excellent prognostic value again. Those N_0_-N_1_ patients with higher TMRS showed significantly worse DFS (HR = 2.278, 95% CI: 1.189–4.346, *p*=0.0135) and OS (HR = 2.982, 95% CI: 1.110–7.519, *p*=0.0303) than those with lower TMRS (Figures 7(a) and 7(b)).

## 4. Discussion

The independent prognostic value of serum tumor markers, such as CEA and CA15-3, was revealed in several previous studies [17, 20, 22]. However, among all these studies, there is little discussion on molecular subtypes of breast cancer and few studies were performed to explore the prognostic value of multiple tumor markers. In our current study, we used X-tile to determine the best prognostic cut-off value of each tumor marker based on the idea of “optimal cut-off value” [36] and confirmed the significant prognostic role of CEA, CA125, and CA211. On the other hand, we synthesized the role of six tumor markers and constructed an excellent prognostic model for stage I-III TNBC patients, providing a method for assisting in predicting prognosis.

Among the six tumor markers included in our study, CEA and CA15-3 were mostly demonstrated and their elevated levels were closely related to poor prognosis in breast cancer patients [17, 20, 22, 23, 38]. Wu SG et al. found that elevated levels of CEA and CA15-3 had no significant effect on local recurrence-free survival but were significantly associated with the decrease of distant metastasis-free survival, DFS, and OS in the Chinese breast cancer cohort [23]. The correlation analysis between molecular subtypes and tumor markers indicated that there was only 1 case (1.6%) in TNBC with elevated CEA, much less than other subtypes, while the proportion of CA15-3 (14.3%) was similar to others [23]. Although two additional studies confirmed the significant prognostic value of CEA and CA15-3 for DFS and OS in overall breast cancer patients, subgroup analysis of molecular subtype showed inconsistent results [20, 38]. The study of Nam SE et al. suggested no correlation between the levels of CEA, CA15-3, and OS of TNBC patients, while another research indicated that in basal-like subtype, which had an overlap of approximately 70–80% TNBC patients, elevated CEA conferred reduction for breast cancer-specific survival (BCSS), but without association observed for DFS [20, 38]. Different from our study, the studies mentioned above all were performed based on the clinical upper limit as the prognostic cut-off. The negative evidence in the TNBC subtype suggested that perhaps we should screen an optimal cut-off used for prognosis. Our results confirmed the prognostic value of CEA in early-stage TNBC patients when using the cut-off selected by X-tile. CA125, which is mostly used in ovarian cancer, was found to increase significantly in metastatic breast cancer patients [39, 40]. In Li JX's study, there was no relevance found between CA125 and breast cancer outcomes, including BCSS and DFS [38]. But another study that included young breast cancer patients indicated that a high level of CA125 was associated with worse DFS and OS when using 19.38 U/mL as the cut-off value [41], providing further evidence for selecting an optimal cut-off value. Although no study explored the prognostic significance of CA125 in different molecular subtypes, it was shown that the levels of CA125 in TNBC patients were higher than non-TNBC, suggesting that elevation of CA125 can be used to predict a poor outcome of TNBC patients [42]. According to our results, CA125 showed a significant prognostic value when using 9.05 U/mL as the cut-off. As for CA19-9, CA242, and CA211, there were quite a few studies exploring their relationship with breast cancer. Some researchers have investigated the diagnostic value of CA19-9 in breast cancer [43], but its role in predicting prognosis still remains unknown. CA242 and CA211 were discovered relatively later than other tumor markers. Thanks to their low specificity, most studies explored its application in the diagnosis or prognosis of pancreatic cancer or gastrointestinal cancer [44, 45]. This is the first time to report the significant prognostic value of CA211 in breast cancer patients, suggesting that its role in breast cancer is worthy of further study.

In the present study, we found that both the lasso Cox model and RSF model based on tumor markers could help stratify stage I-III TNBC patients' recurrence risk and mortality risk. Numerous previous studies adopted the Cox proportional hazard model with lasso penalization for survival data [30, 46] because it had wider application value for its role in simplifying variables. Our results also suggested that lasso Cox model had a larger AUC value and better prognostic significance than the RSF model. Therefore, we developed a prognostic model involving five tumor markers except for CA242, which are easily detected in clinical practice, to calculate TMRS based on machine learning algorithms for predicting the outcome of TNBC patients. The role of tumor markers was further validated in our study. When comparing the clinicopathological characteristics, we found that the high TMRS group indicated a more advanced stage, with more lymph nodes metastasis (Table S1), which provided the possibility of estimating the stage according to tumor markers. In addition to being associated with tumor burdens, TMRS groups were also reported an excellent prognostic significance for TNBC patients. The multivariable analysis also confirmed that TMRS was one of the independent prognostic factors. Thus, the prognostic value of the combination of multiple tumor marker levels was further intensified in our study. Although the ASCO has not recommended therapeutic decisions based on the serum tumor marker status [26], we still think that elevated serum tumor markers could be useful in discriminating high-risk groups, for which the hypothesis should be verified.

There are some limitations to this study that should be considered as well. First of all, we did not verify the validity of the model by using a verification set. Since public datasets that provide information about levels of patients' serum tumor markers are inaccessible, we can not perform further analysis based on an external dataset. On the other hand, it is a single-center study with a limited number of patients, and all the patients included are in the Chinese cohort, so multicenter prospective studies should be performed to confirm the validity of this prognostic model. In addition, due to the generally good prognosis of breast cancer, the number of cases with recurrence or death was small. Given this limitation, longer-term follow-up will be needed to update the results. What is more, we did not evaluate the prognosis of patients by comparing their changes in serum tumor marker concentrations before and after surgery, which is also a strategy of using tumor marker. Finally, whether the prognostic model is suitable for metastatic patients and other molecular subtypes is worthy of further exploration.

In conclusion, our study indicated that pretreatment levels of serum CEA, CA125, and CA211 had great prognostic significance for TNBC patients when using the optimal cut-off value determined by X-tile. TMRS, which was calculated based on tumor markers by taking the lasso Cox model, was an independent prognostic factor as well. A higher score of TMRS was associated with worse DFS and OS both in stage I-III and N_0_-N_1_ TNBC patients. We hope that further study should be used in an effort to confirm the validity of this study and to provide more information by using tumor markers regarding therapeutic decision-making in clinical practice.

## Figures and Tables

**Table 1:** Baseline clinicopathological characteristics of the study population (*n* = 258).

Characteristics	No. of patients	Percent/%
Age at diagnosis/years old (median 51.5)^#^		
<40	22	8.5
40∼50	87	33.7
50∼60	89	34.5
≥60	60	23.3

Side		
Left	131	50.8
Right	126	48.8
Bilateral	1	0.4

Histology		
Nonspecific invasive cancer	203	78.7
Mixed	30	11.6
Other	25	9.7

Grade		
I	6	2.3
II	77	29.9
III	110	42.6
Unknown	65	25.2

Ki-67		
<30%	49	19.0
≥30%	193	74.8
Unknown	16	6.2

T-stage		
1	128	49.6
2	99	38.4
3	16	6.2
4	3	1.2
Unknown	12	4.6

N-stage		
0	183	70.9
1	46	17.8
2	18	7.0
3	11	4.3

Stage		
I	100	38.8
II	111	43.0
III	36	14.0
Unknown	11	4.3

Surgery		
Lumpectomy	80	31.0
Mastectomy	178	69.0

Chemotherapy		
Yes	236	91.5
No	14	5.4
Unknown	8	3.1

Radiotherapy		
Yes	114	44.2
No	124	48.1
Unknown	20	7.7

No. of events total: *n* = 53 (20.5%)		
Locoregional recurrence	16	6.2
Distant metastasis^##^	31	12.0
Second primary cancer	3	1.2
Death because of other reasons	3	1.2

No. of deaths total: *n* = 28 (10.8%)		
Death because of breast cancer	23	8.9
Death because of other reasons	5	1.9

^#^The age range takes the lower limit but not the upper limit. ^##^ Some patients with distant metastasis were accompanied by locoregional recurrence, all of which were counted as distant metastasis.

**Table 2:** The optimal prognostic cut-off value of each tumor marker determined by X-tile.

Tumor marker	Cut-off	No. of patients N (%)	
CEA/(ng/mL)	2.15	<2.15	167 (64.7)
		≥2.15	91 (35.3)
CA19-9/(U/mL)	17.30	<17.30	202 (78.3)
		≥17.30	56 (21.7)
CA125/(U/mL)	9.05	<9.05	64 (24.8)
		≥9.05	194 (75.2)
CA242/(U/mL)	8.85	<8.85	217 (84.1)
		≥8.85	41 (15.9)
CA211/(ng/mL)	1.15	<1.15	155 (60.1)
		≥1.15	103 (39.9)
CA15-3/(U/mL)	16.00	<16.00	215 (83.3)
		≥16.00	43 (16.7)

CEA: carcinoembryonic antigen; CA: cancer antigen.

**Table 3:** Regression coefficients of the lasso Cox model.

Tumor marker	*β* ^*∗*^
CEA	0.270645
CA19-9	0.262472
CA125	0.619252
CA242	0
CA211	0.713767
CA15-3	0.004887

^∗^Positive regression coefficients indicate that higher serum tumor marker levels contributed to higher recurrence risks, while negative coefficients indicate that higher tumor marker levels contributed to lower recurrence risks. CEA: carcinoembryonic antigen; CA: cancer antigen.

**Table 4:** Univariable and multivariable analyses for DFS in stage I-III TNBC patients.

Characteristics	Univariable analysis		*p* value	Multivariable analysis		*p* value
	HR	95%CI		HR	95%CI	
Age at diagnosis						
<40	Ref		0.242	-		
40∼50	0.449	0.184–1.096		-		
50∼60	0.437	0.178–1.075		-		
≥60	0.630	0.257–1.548		-		

Grade						
I	Ref		0.169	-		
II	1.743	0.234–12.953		-		
III	0.917	0.121–6.983		-		

Ki-67						
<30%	Ref		0.646	-		
≥30%	0.857	0.444–1.654		-		

T-stage						
1	Ref		0.093	Ref		0.382
2	1.876	1.037–3.393		1.587	0.842–2.990	
3	2.249	0.764–6.622		2.090	0.686–6.364	
4	1.896	0.254–14.175		2.044	0.268–15.580	

N-stage						
0	Ref		<0.001^∗∗∗^	Ref		<0.001^∗∗∗^
1	0.903	0.396–2.057		0.658	0.263–1.650	
2	3.732	1.705–8.171		2.767	1.218–6.288	
3	7.775	3.547–17.040		4.980	2.081–11.917	

Stage						
I	Ref		<0.001^∗∗∗^	-		
II	1.651	0.816–3.340		-		
III	5.380	2.564–11.288		-		

TMRS groups						
Low TMRS	Ref		<0.001^∗∗∗^	Ref		0.002^∗∗^
High TMRS	3.173	1.718–5.862		2.847	1.473–5.506	

Cox's proportional hazard analysis was carried out for univariable and multivariable analyses to identify independent prognostic factors for DFS in stage I-III TNBC patients. Multivariable analysis was performed further for the factor whose *p* < 0.10 in univariable analysis. ^*∗∗*^*p* < 0.01, ^*∗∗∗*^*p* < 0.001 indicate a significant difference. DFS: disease-free survival; TNBC: triple-negative breast cancer; HR: hazard ratio; CI: confidence interval; TMRS: tumor marker risk score.

## Data Availability

The underlying data used to support our findings of this study are available from the corresponding author on request.

## Abbreviations:

TNBC: Triple-negative breast cancer
ER: Estrogen receptor
PR: Progesterone receptor
HER-2: Human epidermal growth factor receptor-2
pCR: Pathologic complete response
LRR: Locoregional recurrence
DFS: Disease-free survival
OS: Overall survival
BCSS: Breast cancer cause-specific survival
CEA: Carcinoembryonic antigen
CA: Cancer antigen
ASCO: American Society of Clinical Oncology
NCCN: National Comprehensive Cancer Network
HR: Hazard ratio
CI: Confidence interval
TMRS: Tumor marker risk score
ROC: Receiver operating characteristic
AUC: Area under the ROC curve
RSF: Random survival forest.

## Supplementary Materials

Supplementary Materials Figure S1: The levels of each serum tumor marker in patients without event, local recurrence, and distant metastasis. (A) CEA. (B) CA19-9. (C) CA125. (D) CA242. (E) CA211. (F) CA15-3. A scatter represents a patient, and the cut-off value of each scatter plot is the clinical upper limit. The comparison of tumor markers' levels between different groups was performed using one-way ANOVA and Tukey's post hoc test or nonparametric Kruskal-Wallis test as appropriate (CEA: carcinoembryonic antigen; CA: cancer antigen; TNBC: triple-negative breast cancer; NS: not significant). Table S1: Clinicopathological characteristics of the patients according to TMRS.
