# Supplementary material for: Prognostic Models for Nonmetastatic Triple-Negative Breast Cancer Based on the Pretreatment Serum Tumor Markers with Machine Learning
Source: J Oncol. 2021 May 15;2021:6641421. doi: 10.1155/2021/6641421 (PMC8147528; doi:10.1155/2021/6641421)
Supplement: Supplementary Materials — Figure S1: The levels of each serum tumor marker in patients without event, local recurrence, and distant metastasis. (A) CEA. (B) CA19-9. (C) CA125. (D) CA242. (E) CA211. (F) CA15-3. A scatter represents a patient, and the cut-off value of each scatter plot is the clinical upper limit. The comparison of tumor markers' levels between different groups was performed using one-way ANOVA and Tukey's post hoc test or nonparametric Kruskal-Wallis test as appropriate (CEA: carcinoembryonic antigen; CA: cancer antigen; TNBC: triple-negative breast cancer; NS: not significant). Table S1: Clinicopathological characteristics of the patients according to TMRS. [file 6641421.f1.docx]

**Figure S1. The levels of each serum tumor markers in without event, local recurrence and distant metastasis patients.** (A) CEA. (B) CA19-9. (C) CA125. (D) CA242. (E) CA211. (F) CA15-3. A scatter represents a patient, and the cut-off value of each scatter plot is clinical upper limit. The comparison of tumor markers’ levels between different groups was performed using one-way ANOVA and Tukey post-hoc test or nonparametric Kruskal-Wallis test as appropriate. (CEA: carcinoembryonic antigen; CA: cancer antigen; TNBC: triple-negative breast cancer; NS: not significant)

Table S1. Clinicopathological characteristics of the patients according to TMRS

| Characteristics | No. of patient (%) | | *p* value |
| --- | --- | --- | --- |
|  | Low TMRS  (n=125) | High TMRS  (n=133) |  |
| Age at diagnosis | | | |
| <40 | 11 (8.8) | 11 (8.3) | 0.006^**^ |
| 40~50 | 54 (43.2) | 33 (24.8) |  |
| 50~60 | 40 (32.0) | 49 (36.8) |  |
| ≥60 | 20 (16.0) | 40 (30.1) |  |
| Side | | | |
| Left | 62 (49.6) | 69 (51.9) | 0.560 |
| Right | 62 (49.6) | 64 (48.1) |  |
| Bilateral | 1 (0.8) | 0 (0) |  |
| Histology | | | |
| Non-specific invasive cancer | 97 (77.6) | 106 (79.7) | 0.850 |
| Mixed | 16 (12.8) | 14 (10.5) |  |
| Other | 12 (9.6) | 13 (9.8) |  |
| Grade | | | |
| I | 5 (4.0) | 1 (0.8) | 0.128 |
| II | 36 (28.8) | 41 (30.8) |  |
| III | 58 (46.4) | 52 (39.1) |  |
| Unknown | 26 (20.8) | 39 (29.3) |  |
| Ki-67 | | | |
| <30% | 23 (18.4) | 26 (19.6) | 0.624 |
| ≥30% | 96 (76.8) | 97 (72.9) |  |
| Unknown | 6 (4.8) | 10 (7.5) |  |
| T-stage | | | |
| 1 | 65 (52.0) | 63 (47.4) | 0.056 |
| 2 | 51 (40.8) | 48 (36.1) |  |
| 3 | 2 (1.6) | 14 (10.5) |  |
| 4 | 1 (0.8) | 2 (1.5) |  |
| Unknown | 6 (4.8) | 6 (4.5) |  |
| N-stage | | | |
| 0 | 104 (83.2) | 79 (59.4) | <0.001^***^ |
| 1 | 15 (12.0) | 31 (23.3) |  |
| 2 | 4 (3.2) | 14 (10.5) |  |
| 3 | 2 (1.6) | 9 (6.8) |  |
| Stage | | | |
| I | 57 (45.6) | 43 (32.3) | 0.002^**^ |
| II | 56 (44.8) | 55 (41.4) |  |
| III | 7 (5.6) | 29 (21.8) |  |
| Unknown | 5 (4.0) | 6 (4.5) |  |
| Surgery | | | |
| Lumpectomy | 44 (35.2) | 36 (27.1) | 0.158 |
| Mastectomy | 81 (64.8) | 97 (72.9) |  |
| Chemotherapy | | | |
| Yes | 115 (92.0) | 121 (91.0) | 0.461 |
| No | 5 (4.0) | 9 (6.8) |  |
| Unknown | 5 (4.0) | 3 (2.2) |  |
| Radiotherapy | | | |
| Yes | 52 (41.6) | 62 (46.6) | 0.423 |
| No | 65 (52.0) | 59 (44.4) |  |
| Unknown | 8 (6.4) | 12 (9.0) |  |

The chi-square or Fisher’s exact test was performed as appropriate to evaluate the difference between low-score and high-score group. ***p*<0.01, ****p*<0.001, indicate a significant difference. (TMRS: tumor marker risk score)
